# Imputation of Missing Data for Time-to-Event Endpoints Using Retrieved Dropouts

**DOI:** 10.1007/s43441-023-00575-5

**Published:** 2023-10-07

**Authors:** Shuai Wang, Robert Frederich, James P. Mancuso

**Affiliations:** 1 Pfizer Inc., 1 Portland St, Cambridge, MA 02139 USA; 2 Pfizer Inc., 66 Hudson Blvd, New York, NY 10001 USA; 3 Pfizer Inc., 280 Shennecossett Rd, Groton, CT 06340 USA

**Keywords:** Time to event, Missing data, Multiple imputation, Cardiovascular outcome trial, Estimand, Informative censoring, Proper imputation

## Abstract

We have explored several statistical approaches to impute missing time-to-event data that arise from outcome trials with relatively long follow-up periods. Aligning with the primary estimand, such analyses evaluate the robustness of results by imposing an assumption different from censoring at random (CAR). Although there have been debates over which assumption and which method is more appropriate to be applied to the imputation, we propose to use the collection of retrieved dropouts as the basis of missing data imputation. As retrieved dropouts share a similar disposition, such as treatment discontinuation, with subjects who have missing data, they can reasonably be assumed to characterize the distribution of time-to-event among subjects with missing data. In terms of computational intensity and robustness to violation of underlying distributional assumption, we have compared parametric approaches via MCMC or MLE multivariate sampling procedures to a non-parametric bootstrap approach with respect to baseline hazard function. Each of these approaches follows a process of multiple imputation (“proper imputations”), analysis of complete datasets, and final combination. The type-I error, and power rates are examined under a wide range of scenarios to inform the performance characteristics. A subset of a real unblinded phase III CVOT is used to demonstrate the application of the proposed approaches, compared to the Cox proportional hazards model and jump-to-reference multiple imputation.

**Supplementary Information:**

The online version contains supplementary material available at 10.1007/s43441-023-00575-5.

## Introduction

In the 2008 published regulatory guidance [1] for type 2 diabetes medications, cardiovascular outcome trials (CVOTs) were required to demonstrate that the investigational drug would not increase the risk of major adverse cardiovascular events by 30% or more, compared to placebo. All subsequent CVOTs in this field to date have met this requirement and the regulatory agency is in the process of rolling out a new guidance [2] to replace the 2008 version, with a new focus on size of the safety trial and patient population characteristics. These safety trials feature very large sample size, long duration and consequently come at very high costs, aiming to rule out cardiovascular risk increase and potentially to show cardiovascular or renal benefits. Beyond diabetes, CVOTs are also commonly conducted in lipid-lowering therapies [3, pp. 1713–1722, 4, pp. 2387–2397, 5, pp. 1527–1539]. Powered by even larger sample size and higher number of events to accrue, CVOTs for lipid-lowering drugs usually aim to demonstrate cardiovascular benefits.

In most CVOTs, time to event endpoints are primarily analyzed using a semi-parametric Cox proportional hazards (CPH) model in which discontinued subjects with no prior events are censored at their study discontinuation date, assuming non-informative censoring [6, pp. 139–156], usually justifiable by censoring at random in each treatment group [7, pp. 1–6, 8]. Although every study aims to keep all participants in the study, some subjects will prematurely discontinue study for various reasons including withdrawal of consent, lost to follow-up, physician/sponsor decision, subject move, site closure, etc.

Missing data can be generally defined as subjects who prematurely discontinue the study with no prior events that can be counted towards the endpoint of interest. If the endpoint of interest has already been reached, followed by the subject’s discontinuation from the study, then that would not be considered missing data according to this definition***.*** As most CVOTs aim to follow subjects from randomization until death or end of study, whichever occurs first, deaths occurring during the study will generally not be considered as missing data. For example, consider a scenario in which a subject dies during the study and the endpoint is time to first occurrence of 3-point MACE (defined as a composite of non-fatal MI, non-fatal stroke and CV death). If this subject has died due to CV cause, he/she will not be counted as missing data because his/her CV death is counted as a MACE event. In the same example, if he/she dies due to non-CV cause and he/she has no MACE events prior to death, he/she will be counted as complete data with no event, and a time-to-event will not be imputed. Overall, it’s not trivial to handle death data because death events often come with limited data on which to adjudicate the cause. Because missing data censored at random in CPH model can potentially result in a biased estimate [9, p. 4, 10, p. 40], we take a missing not at random (MNAR) approach to impute the time to event/censoring based on observed discontinuation and then present a combined estimate of treatment effect.

Missing data sensitivity analyses have become more and more essential to support robustness of primary conclusions, with regulatory agencies seeking for consistency with primary conclusions [11]. Overall, a lot of progress has been made in the past decade on continuous endpoints, with various methods based on different MNAR assumptions proposed. For instance, return to baseline [12, pp. 242–248, 13, pp. 641–653] assumes discontinued subjects will experience a washout effect and hence eventually return to their baseline levels. The class of control-based methods [14, pp. 1352–1371, 15, pp. 443–463] assumes subjects who discontinue the test treatment will follow the distribution of the reference group after discontinuation. Stress testing strategies such as tipping point analyses [16, pp. 1085–1098] is another very popular approach that has gained much attention in recent years. It searches for the breaking point under which statistical significance will vanish. These sensitivity analysis approaches can be adapted and readily applied to binary endpoints due to the connection between linear model and generalized linear model theory. Sensitivity analyses for time to event endpoints are, nevertheless, underdeveloped. There have been some key contributors in this area in the past decade. For instance, Jackson et al. [17, pp. 4681–4694] was the pioneer to propose a non-parametric bootstrap approach for imputation of missing data based on non-independent censoring (i.e., censoring not at random) and Ruau et al. implemented his proposed approach in an R package [18]. Because Jackson’s approach used a step function to estimate the cumulative hazard function $H(t)$, imputed time to event can only take the value of one of the observed times to event. As a result, his approach is not a full estimation approach and will not be applicable to a dataset with very few events due to the nature of the proposed procedures via step functions. Lipkovich et al. [19], pp. 216–229] treated missing data as non-ignorable and imputed them under tipping point framework (delta-adjusted), using piecewise exponential distribution and bootstrap sampling respectively. However, there have been no rules proposed in terms of which deltashould be selected for a standalone sensitivity analysis. It’s noteworthy that both Jackson and Lipkovich described the application of delta/gamma approach which serves as the foundation for tipping point sensitivity analysis. Zhao et al. [20, pp. 229–253] proposed linear interpolation to conduct imputation based on bootstrap sampling, using Kaplan–Meier (KM) and Cox proportional hazards model respectively. Because the field lacks a generalizable overall MNAR (or informative censoring) assumption that can be directly applied to missing data imputation, all above approaches use the full dataset as the imputation basis and heavily rely on a user-specified delta/gamma adjustment to achieve MAR or MNAR imputation.

In the era of estimands when more and more sponsors integrate estimands into the protocol framework, it dictates the need to align proposed sensitivity analyses with primary analyses on estimands, according to ICH E9 R1 [21]. In other words, after primary analysis and primary estimand are pre-specified in the protocol, the proposed sensitivity analyses must target the same estimand so that the results can be compared side by side. Because analyses of time to first events in CVOT are usually conducted under the intent to treat (ITT) principle with no exclusion of data post randomization, following the treatment policy (TP) strategy [22, pp. 1–19] there is no controversy that all subsequent sensitivity analyses should target the same TP estimand, in terms of handling of missing data that arise from intercurrent events.

It’s not always easy to reach common ground between sponsors and regulators on the choice of sensitivity analyses. When sponsors propose a sensitivity analysis based on missing at random (MAR), regulatory agencies are interested in knowing if the primary conclusion will still hold after imposing some unfavorable assumed conditions. Therefore, it’s critical and urgent to settle on something that’s relatively acceptable to both parties. Assuming MAR for all discontinued subjects can be criticized, especially for subjects who discontinued due to reasons that might be plausibly related to their treatment (e.g., side effects), but imposing the worst assumptions on all missing data is also hard to justify, and ultimately, the choice of assumptions for imputing missing data should be driven by the estimand of interest. The approach we propose can serve as a balance point between sponsor and regulatory agencies because its underlying MNAR assumption is quite neutral. The assumption states that subjects with missing data will have similar treatment effect compared to retrieved dropouts (defined as off-treatment subjects that remain in the study) in the same randomized group. It uses the subpopulation of “off-treatment” subjects to multiply impute the missing data.

Recently He et al. published a paper about retrieved dropout multiple imputation (MI) in CVOT using piecewise exponential distribution [22, pp. 1–19]. It was demonstrated using a lipid-lowering CVOT dataset, with respect to time to first MACE. Results of the piecewise exponential model were compared to the regular CPH model and the jump to reference multiple imputation. However, simulations were not conducted to inform type-I error and power rates. In our manuscript, we explore the approach in different ways: parametrically or non-parametrically with respect to the baseline hazard function. More specifically, we explore bootstrap, piecewise exponential and Weibull distributions for the imputation of time to event missing data. Simulation studies are conducted to help understand the type-I error and power rates under different clinical trial scenarios. Lastly, we illustrate these approaches by applying them to a real unblinded phase 3 CVOT.

The CVOT dataset needs to be broken down into 3 non-overlapping subsets, before the initiation of the analysis. Similar to the implementations in continuous endpoints [23, p. 82], retrieved dropouts (RDs) in outcome trials are usually defined as subjects who have discontinued randomized treatment but continue to stay in the trial. Because they are continuously followed until the occurrence of death or administrative censoring datacut, their outcome will be known. In the proposed analyses, they form the fundamental basis of missing data imputation, given that they resemble the missing data subjects in terms of treatment discontinuation and given the assumption that subjects who discontinued treatment will have similar outcomes whether they were followed up in the study or not. Subjects who have withdrawn consent or lost to follow-up will not take the assigned study medication, which is essentially identical to RDs except that RDs continue to stay in the study so that their outcome will be known. Completers, on the other hand, are defined as subjects who have completed the study while on study medication. Missing data is the remainder of the CVOT dataset, i.e., subjects who have discontinued from the study with no prior endpoint events. In the “Statistical Methods” section, we will first illustrate these methods with mathematical notation. Then we will summarize the implementation into an algorithm format. Next, we will conduct simulation studies to understand and compare the performance of each approach under different clinical trial scenarios. The data analysis section provides a real application of these analyses to an unblinded CVOT dataset. Finally, all findings will be discussed and concluded.

## Statistical Methods

### The Basis of Time to Event Imputation

Assume in a CVOT $2N$ subjects are randomized to two treatment groups in a 1:1 allocation ratio. Let $T={T}_{i} (i=1,\dots , 2N)$ denote the time to first event/censoring of the primary endpoint. Then ${E}_{i}$ is used to denote the event status: they are assigned a value of 1 for events and 0 otherwise.

We first model the conditional probability of a subject with missing data because this will enable the derivation of imputed time to first event. Let $c$ denote the observed time to censoring, e.g. from randomization to study discontinuation, $t$ is the unknown time to first event. $S\left(t\right)$ stands for the survival function of $T$, then we have the following general equation:

1 $$P\left(T\le t|T>\mathrm{c}\right)=1-P\left(T>t|T>\mathrm{c}\right)=1-\frac{S\left(t\right)}{S\left(\mathrm{c}\right)}\in \left(\mathrm{0,1}\right).$$

$t$ needs to satisfy the condition that$t>c$, otherwise the above equation will reduce to 0. Then based on the above equation, we can randomly sample a variable from uniform distribution, i.e. $u\sim unif(\mathrm{0,1})$ and $t$ can be estimated by solving the equation$\frac{S\left(t\right)}{S\left(\mathrm{c}\right)}=u$. Due to the monotone property of$S\left(t\right)$, the estimated $\widehat{t}$ is ensured to be greater than$\mathrm{c}$. Similarly this can be applied to all $k$ subjects with missing data, by drawing k independent variables (${u}_{1}$, …,${u}_{k}$) from $unif(\mathrm{0,1})$, s.t.

2 $$\frac{{S}_{j}\left({t}_{j}\right)}{{S}_{j}\left({\mathrm{c}}_{\mathrm{j}}\right)}={u}_{j} \left(j=1,\dots , k\right),$$

where ${S}_{j}\left({\mathrm{c}}_{\mathrm{j}}\right)=S\left({\mathrm{c}}_{\mathrm{j}}|{{trt}_{j}, {\varvec{X}}}_{j}\right)=P\left(T>{\mathrm{c}}_{\mathrm{j}}|{{trt}_{j}, {\varvec{X}}}_{j}\right)$ are survival functions adjusting for treatment and baseline covariates and ${\widehat{t}}_{j}$ are the estimates of ${t}_{j}$ by solving Eq. (2). For simplicity with no loss of generalization, let ${trt}_{j}=1$ denote active treatment group and ${trt}_{j}=0$ denote placebo group and ${{\varvec{X}}}_{j}$ is the vector of baseline covariates.

Next, we will describe how we combine this imputation step with the CPH model when the baseline hazard function ${h}_{0}(t$) is derived using: (1) non-parametric bootstrap sampling, (2) parametric distribution.

### Bootstrap

When an explicit parametric form is not imposed on baseline hazard function ${h}_{0}(t$), just like in the semi-parametric Cox proportional hazards model, we propose to use bootstrap and interpolation to multiply impute the time to event conditional on observed time to censoring. Let ${C}_{j}$ denote the individual time to study closure from randomization ${R}_{j}$. Without loss of generalization, assume the study uses a common study datacut *D* and ${C}_{j}$ is defined as ${C}_{j}=D-{R}_{j}+1$ in days.

Assume there are a total of B bootstrap samples which generate B sets of survival function. Given a bootstrap sample $b$, the associated survival function is defined as ${S}_{j}^{\left(b\right)}\left(t\right)={S}^{(b)}\left(t|{{trt}_{j}, {\varvec{X}}}_{j}\right)={P}^{(b)}\left(T>t|{{trt}_{j}, {\varvec{X}}}_{j}\right)$ ($b=1, \dots , B$). We first construct a grid with (m + 2) grid points as follows: ${\mathrm{c}}_{\mathrm{j}}={t}_{j}^{\left(0\right)}< {t}_{j}^{\left(1\right)}< {t}_{j}^{\left(2\right)}<\dots <{t}_{j}^{\left(m-1\right)}< {t}_{j}^{\left(m\right)}<{t}_{j}^{\left(m+1\right)}={\mathrm{C}}_{\mathrm{j}}$. Then ${S}_{j}^{\left(b\right)}\left({c}_{j}\right), { S}_{j}^{\left(b\right)}\left({t}_{j}^{\left(1\right)}\right),\dots ,{ S}_{j}^{\left(b\right)}\left({\mathrm{C}}_{\mathrm{j}}\right)$ can be derived using “predict” function along with “coxph” in R, written as ${\widehat{S}}_{j}^{\left(b\right)}\left({c}_{j}\right), \dots , {\widehat{S}}_{j}^{\left(b\right)}\left({\mathrm{C}}_{j}\right)$. Since ${S}_{j}^{\left(b\right)}\left({t}_{j}\right)$ is derived as ${\widehat{S}}_{j}^{\left(b\right)}\left({t}_{j}\right)={S}_{j}^{\left(b\right)}\left({c}_{j}\right){{u}_{j}}^{(b)}$ according to Eq. (2), we can identify interval, say (${t}_{j}^{\left(p\right)}, {t}_{j}^{\left(p+1\right)})$ in which ${{t}_{j}}^{(b)}$ falls, s.t. ${\widehat{S}}_{j}^{\left(b\right)}\left({t}_{j}^{\left(p+1\right)}\right)\le   {\widehat{S}}_{j}^{\left(b\right)}\left({t}_{j}\right)\le { \widehat{S}}_{j}^{\left(b\right)}\left({t}_{j}^{p}\right))$. Then ${t}_{j}$ is estimated using linear interpolation, i.e.

3 $${{\widehat{t}}_{j}}^{(b)} ={t}_{j}^{\left(p\right)}+\frac{{t}_{j}^{\left(p+1\right)}-{t}_{j}^{p}}{{ \widehat{S}}_{j}^{\left(b\right)}\left({t}_{j}^{\left(p+1\right)}\right)-{ \widehat{S}}_{j}^{\left(b\right)}\left({t}_{j}^{p}\right)}\left({ \widehat{S}}_{j}^{\left(b\right)}\left({t}_{j}\right)-{ \widehat{S}}_{j}^{\left(b\right)}\left({t}_{j}^{p}\right)\right).$$

If ${{t}_{j}}^{(b)}$ falls outside the grids, then ${{t}_{j}}^{(b)}$ is estimated as ${{\widehat{t}}_{j}}^{(b)}={\mathrm{C}}_{\mathrm{j}}$.

### Parametric

Using parametric distribution to model ${h}_{0}(t)$, estimates of ${t}_{j}$ can be derived in closed form. In the following sections, we demonstrate how to use piecewise exponential and Weibull distributions respectively to model the distribution of ${h}_{0}(t)$ because they are able to represent distribution of most baseline hazard functions for time to event endpoints [24, pp. 682–701, 25, p. 873, 26, pp. 59–73, 27, pp. 10–11, 28, p. 152].

#### Piecewise Exponential

When baseline hazard function follows a piecewise exponential distribution, the proportional hazards model is written as $h\left(t|{ {\varvec{Z}}}_{j};{\varvec{\theta}}\right)={h}_{0}(t){e}^{{\boldsymbol{ }{\varvec{Z}}}_{{\varvec{j}}}{\varvec{\beta}}}$ where ${h}_{0}\left(t\right)={\lambda }_{u}$ for $t$ in $[{\tau }_{u-1}, {\tau }_{u})$, with 0 = ${\tau }_{0}<{\tau }_{1}<\dots <{\tau }_{M}=\infty$ as partitioning points to be estimated as well and ${{\varvec{Z}}}_{j}$ is the full design vector denoted as ${{\varvec{Z}}}_{j}=({{trt}_{j}, {\varvec{X}}}_{j})$. Its corresponding survival function is written as

4 $$S\left(t|{ {\varvec{Z}}}_{j};{\varvec{\theta}}\right)=\left\{\begin{array}{cc}\mathrm{exp}(-{\lambda }_{1}t{e}^{{\boldsymbol{ }{\varvec{Z}}}_{{\varvec{j}}}{\varvec{\beta}}})& t \epsilon [0, {\tau }_{1})\\ \mathrm{exp}(-({\sum }_{v=1}^{u-1}{\lambda }_{v}({\tau }_{v}-{\tau }_{v-1})+{\lambda }_{u}\left(t-{\tau }_{u-1}\right)){e}^{{\boldsymbol{ }{\varvec{Z}}}_{{\varvec{j}}}{\varvec{\beta}}})& t \epsilon [{\tau }_{u-1}, {\tau }_{u})\\ \mathrm{exp}(-({\sum }_{v=1}^{M-1}{\lambda }_{v}({\tau }_{v}-{\tau }_{v-1})+{\lambda }_{M}\left(t-{\tau }_{M-1}\right)){e}^{{\boldsymbol{ }{\varvec{Z}}}_{{\varvec{j}}}{\varvec{\beta}}})& t \epsilon [{\tau }_{M-1}, \infty ) \end{array}.\right.$$

A total of B imputations are planned. To make sure the imputation follows the “proper” principle [29, pp. 202–243, 30, pp. 473–489], different sets of coefficients and variance estimates have to be generated and applied to each imputation. The following two methods can fulfill this goal because they are asymptotically equivalent [31, pp. 593–607]. For SAS users, the most convenient way is to use “MCMC sampling” to draw Bayesian posterior samples for parameter coefficients ${\varvec{\beta}}$ and scale parameters ${\lambda }_{u}$ ($u=1, \dots , M)$. The alternative is to draw from a multivariate normal distribution with the MLE estimates as the mean and the estimated variance matrix of the MLE estimates as the variance (i.e., “MLE multivariate sampling”). Both approaches start from fitting a CPH model based on a piecewise exponential distribution using data of all RD subjects, with parameter estimates and estimate of the covariance matrix denoted as $\widehat{{\varvec{\theta}}}=(\widehat{{\varvec{\lambda}}},\boldsymbol{ }\widehat{{\varvec{\beta}}})$ and $\widehat{var}(\widehat{{\varvec{\theta}}})$.

For each imputation $(b=1, \dots , B)$, parameters ${{\varvec{\theta}}}^{(b)}=\left({{\varvec{\lambda}}}^{\left(b\right)}, {{\varvec{\beta}}}^{\left(b\right)}\right)=\left({{\lambda }_{1}}^{\left(b\right)}, \dots , {{\lambda }_{M}}^{\left(b\right)}, {{\varvec{\beta}}}^{\left(b\right)}\right)$ can be randomly sampled using either approach:

- *MCMC sampling:* ${{\lambda }_{m, (t+1)}}^{\left(b\right)}\sim P({\lambda }_{m}|{{\lambda }_{1, \left(t+1\right)}}^{\left(b\right)}, \dots , {{\lambda }_{(m-1), \left(t+1\right)}}^{\left(b\right)}{{, {{\lambda }_{(m+1), \left(t\right)}}^{\left(b\right)}, \dots , \lambda }_{M, \left(t\right)}}^{\left(b\right)}, {{{\varvec{\beta}}}_{ \left(t\right)}}^{\left(b\right)})$ for $m=1, \dots , M$ and continue through ${\varvec{\beta}}$ until iteration *t* is large enough and chain convergence is achieved.
- *MLE multivariate sampling:* sample ${{\varvec{\theta}}}^{(b)}\sim MVN(\widehat{{\varvec{\theta}}},\boldsymbol{ }\widehat{var}\left(\widehat{{\varvec{\theta}}}\right))$.

Then by combining the above survival function formula (4) with (2) and by letting ${S}_{j}^{\left(b\right)}\left(t\right)$ be defined as ${S}_{j}^{\left(b\right)}\left(t\right)=S\left(t|{{\varvec{Z}}}_{j}; {{\varvec{\theta}}}^{(b)}\right)$, the estimated time to first event is expressed as

5 $${{\widehat{t}}_{j}}^{(b)}=\left\{\begin{array}{cc}{\tau }_{M-1}-\frac{\mathrm{log}\left({{u}_{j*}}^{(b)}\right){e}^{{-{\varvec{Z}}}_{{\varvec{j}}}{{\varvec{\beta}}}^{\left(b\right)}}+{\sum }_{v=1}^{M-1}{{\lambda }_{v}}^{\left(b\right)}\left({\tau }_{v}-{\tau }_{v-1}\right)}{{{\lambda }_{M}}^{\left(b\right)}}& {{u}_{j*}}^{(b)}={S}_{j}^{\left(b\right)}\left({c}_{j}\right){{u}_{j}}^{(b)} \in \left( 0 , {S}_{j}^{\left(b\right)}\left({\tau }_{M-1}\right)\right]\\ {\tau }_{p-1}-\frac{\mathrm{log}\left({{u}_{j*}}^{(b)}\right){e}^{{-{\varvec{Z}}}_{{\varvec{j}}}{{\varvec{\beta}}}^{\left(b\right)}}+{\sum }_{v=1}^{p-1}{{\lambda }_{v}}^{\left(b\right)}\left({\tau }_{v}-{\tau }_{v-1}\right)}{{{\lambda }_{p}}^{\left(b\right)}}& {{u}_{j*}}^{(b)} \in \left( {S}_{j}^{\left(b\right)}\left({\tau }_{p}\right) , {S}_{j}^{\left(b\right)}\left({\tau }_{p-1}\right)\right] ; 2\le p<\left(M-1\right).\\ \frac{-\mathrm{log}\left({{u}_{j*}}^{(b)}\right){e}^{{-{\varvec{Z}}}_{{\varvec{j}}}{{\varvec{\beta}}}^{\left(b\right)}}}{{{\lambda }_{1}}^{\left(b\right)}}& {{u}_{j*}}^{(b)}\in ( {S}_{j}^{\left(b\right)}\left({\tau }_{1}\right) , 1]\end{array}\right.$$

#### Weibull

We first introduce accelerated failure time (AFT) [32, pp. 31–51, 33, pp. 1871–1879] model because the following AFT model is directly linked to Weibull regression. AFT model is very frequently used in real applications with statistical packages and software readily available [34, pp. 583–592].

$${\text{log}}{T}_{j}={\gamma }_{0}+{\boldsymbol{ }{\varvec{Z}}}_{{\varvec{j}}}{\varvec{\gamma}}+\sigma {\varepsilon }_{j},$$

where $\sigma$ is scale parameter and ${\varepsilon }_{j}$ follows a Gumbel distribution (also known as extreme-value distribution).

Using the notations of Weibull AFT model, the hazard function based on Weibull distribution can be written as $h\left(t|{ {\varvec{Z}}}_{j}\right)=\frac{1}{\sigma }{{t}^{\frac{1}{\sigma }-1}e}^{-{\boldsymbol{ }({\varvec{Z}}}_{{\varvec{j}}}{\varvec{\gamma}}+{\gamma }_{0})/\sigma }={h}_{0}\left(t\right){e}^{{\boldsymbol{ }{\varvec{Z}}}_{{\varvec{j}}}{\varvec{\beta}}}$ where

$${\varvec{\beta}}=-\frac{{\varvec{\gamma}}}{\sigma },$$

6 $${h}_{0}\left(t\right)=\frac{1}{\sigma }\frac{{t}^{\frac{1}{\sigma }-1}}{{e}^{\frac{{\gamma }_{0}}{\sigma }}}.$$

Therefore, the baseline hazard function follows a Weibull distribution with $\frac{1}{\sigma }$ as the shape parameter and ${e}^{{\gamma }_{0}}$ as the scale parameter. Because the above AFT model is readily implemented in statistical software, e.g., “proc lifereg” in SAS software and “SurvRegCensCov” package in R, we will use notations of AFT model to derive imputed time to event as follows. To start with, we use $\theta =\left(\sigma , {\gamma }_{0},{\varvec{\gamma}}\right)$ to denote the vector of parameters.

For each imputation $(b=1, \dots , B)$, ${{\varvec{\theta}}}^{(b)}$ is sampled from either MCMC or MLE sampling. Then using $S\left(t|{ {\varvec{Z}}}_{j}\right)={e}^{-\mathrm{exp}(-{\eta }_{j}/\sigma {)t}^{\frac{1}{\sigma }}}$ with linear predictor ${\eta }_{j}=\boldsymbol{}{\varvec{Z}}_{{j}}{\varvec{\gamma}}+{\gamma }_{0}$ in conjunction with (2), time to first event is derived as

7 $${{\widehat{t}}_{j}}^{(b)}={({{c}_{j}}^{\frac{1}{{\widehat{\sigma }}^{\left(b\right)}}}-\mathrm{exp}\left(\frac{{\widehat{\eta }}_{j}^{\left(b\right)}}{{\widehat{\sigma }}^{\left(b\right)}}\right)\mathrm{log}\left({{u}_{j}}^{\left(b\right)}\right))}^{{\widehat{\sigma }}^{\left(b\right)}}$$

where $${\widehat{\eta }}_{j}^{\left(b\right)}={\boldsymbol{ }{\varvec{Z}}}_{{j}}{\widehat{{\varvec{\gamma}}}}^{(b)}+{\widehat{{\gamma }_{0}}}^{(b)}.$$

## Algorithm

We summarize the general framework of implementation in this section.

A total of B multiple imputations/bootstrap samples are planned for imputation of missing data.

1. A Cox proportional hazards model is fit to the dataset comprised RDs (“RD dataset”) adjusting for treatment group and other pre-specified baseline covariates.
2. *Bootstrap:* B different input datasets are created, by sampling from RD dataset with replacements. Then B different CPH models are fit, and each model will serve as the basis of the imputation.
3. *Parametric:* One CPH model is fit, using the RD dataset. Then B different sets of parameters are sampled using either Bayesian MCMC sampling or MLE multivariate sampling (i.e., “proper imputation”).
  *For each imputation (b = 1,…, B), proceed to step 2–4 sequentially.*
4. Missing data of time to first event will be estimated using
  *Bootstrap:* Use formula (3) in conjunction with (2) with pre-specified grid points.
  *Parametric:* Use formula (5)/(7) in conjunction with (2).
5. Then imputed time to first event will be added to the randomization date to derive the imputed outcome.
  If the value is greater than the censoring data cut, then the imputed outcome will be deemed as a censored and his time to censoring will be derived as censoring data cut—randomization date + 1 in days.
  Otherwise, the imputed outcome will be deemed as an event and the imputed value will be used as the time to first event. *For studies that conduct vital search on subjects that are lost to follow up or have withdrawn consent, additional steps can be considered*. For instance, if final vital status is death, the imputed event date also needs to be compared to death date. More specifically,
    It will be imputed as an event if and only if the imputed event date + randomization date doesn’t exceed the earlier of death date and study data cut.
    Otherwise, it will be treated as censored and time to censoring will be derived as the earlier of death date and study data cut-randomization date + 1.
6. All imputed outcomes will be analyzed together with RDs and completers using the regular CPH model. Coefficient estimates of treatment effect and standard error (SE) will be generated.
7. Repeat step 2–4 for all B imputations. Coefficient estimates and SE are pooled and combined into a single estimate following Rubin’s rules [29, pp. 202–243].

## Simulation Studies

Simulation studies are conducted to evaluate the validity of the proposed approaches with respect to different clinical trial scenarios. Starting from type-I error simulations we will first present and compare type-I error rate of the three proposed methods, under the null hypothesis that there is no difference of treatment effect between the two randomized groups, i.e. ${H}_{0}: {\text{HR}}=1$ and with respect to different combinations of sample size, discontinuation rate and retrieved dropout rate, then we move to power simulations to evaluate how powerful these approaches are when the alternative hypothesis that the drug is superior to placebo is true, i.e. ${H}_{1}: {\text{HR}}<1$.

### Type-I Error

Five thousand datasets are simulated under the null hypothesis ${H}_{0}: {\text{HR}}=1$. Under each replicate, the trial is simulated as a two-arm randomized clinical trial with a 1:1 allocation ratio, assuming an overall event rate of 2% per person-year according to a few completed CVOT studies of 5–6 year duration [3, pp. 1713–1722, 5, pp. 1527–1539], a 1-year enrollment, a fixed duration of 5.5 years (i.e. a common censoring data cut of 5.5 years from the first randomization is applied). The following 27 scenarios with respect to different sample size, proportion of RDs and missing rate are explored:

- Sample size: 1000, 5000, 10,000;
- RDs: 5%, 20%, 40%;
- Overall discontinuation from the study, by EOS: 5%, 15%, 30%.

Time to event is simulated under exponential distribution. To test how robust the conclusions will hold, an additional distributional assumption is used. Time to discontinuation is simulated independently of events to avoid bias. RDs are randomly selected from the population set excluding study discontinuations to meet the definition and therefore there’s no overlap between *RDs* and *Overall discontinuation from the study*. Empirical type-I error is defined as proportion of simulations significant at a significance level of two-sided 0.05. Results of type-I error are summarized in Table 2 and Supplementary Table 1.

### Power

A thousand datasets are simulated under the alternative hypothesis that the drug is superior to placebo with a 20% risk reduction, i.e. ${H}_{1}: {\text{HR}}=0.8$. The event rate in the placebo group is simulated as 2% per person-year, and the event rate in the active treatment group is modeled as 2% $\times$ ${\text{HR}}$ accordingly, with the exception that RDs in the active group is simulated as 2% $\times$ ${\text{HR}}$* (${\text{HR}}<{\text{HR}}$* < 1) assuming RDs in the active group will have a slightly reduced risk reduction due to off-treatment periods. ${\text{HR}}$* = 0.9 is used in this instance. The placebo discontinuation rate is simulated in the same way as in type-I error simulations. Whereas, the active group discontinuation rate is assumed to be lower by 2%, compared to placebo. Scenarios with sample size of 1000 is removed because a CVOT with 1000 subjects is very underpowered. The other parameters used in simulations remain the same as type-I error simulations.

**Figure 1:**
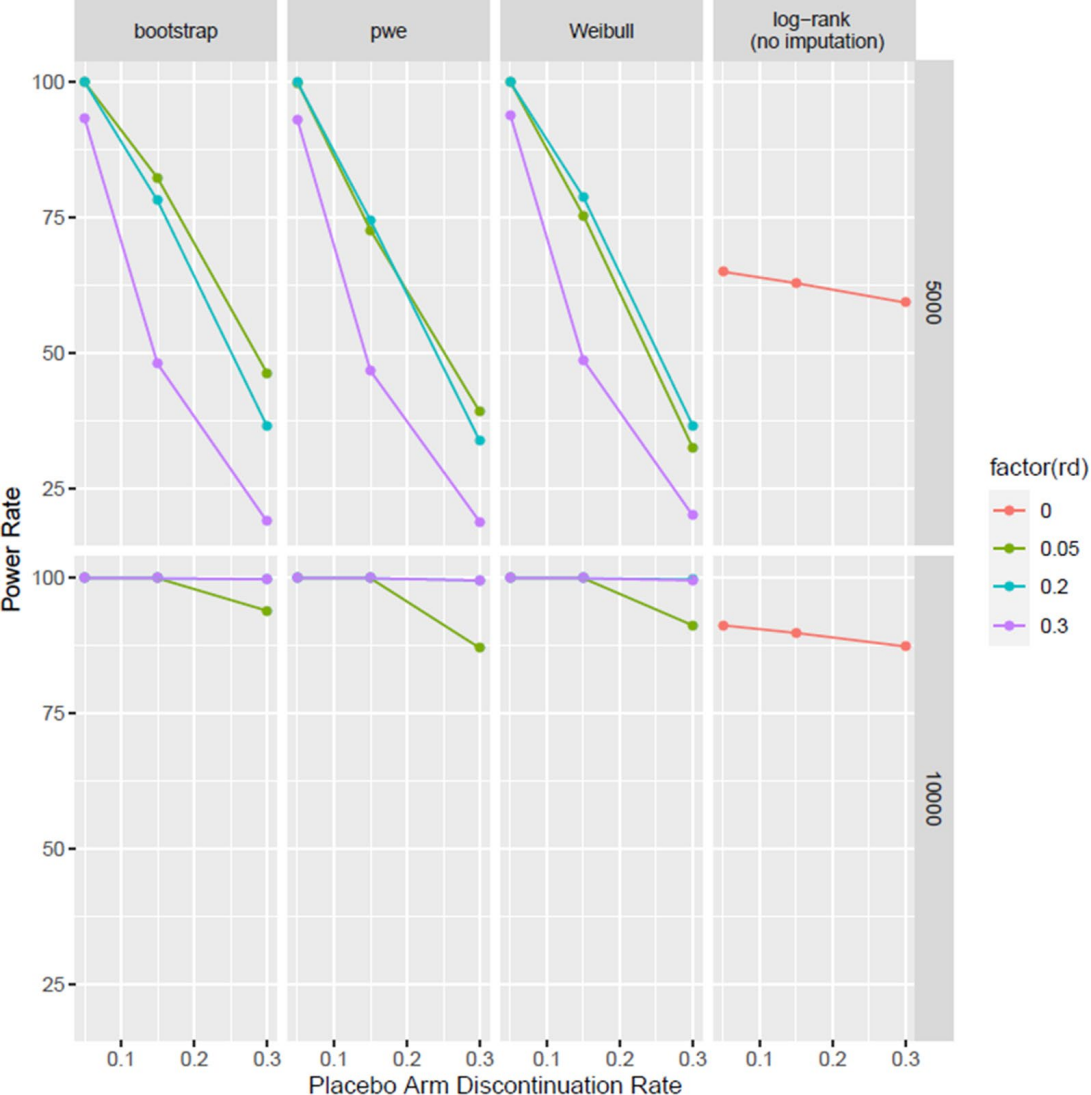
Compare the Power Rate of Three MIRD Approaches Under Different Clinical Scenarios

Empirical power rate is defined as proportion of simulations significant at a significance level of two-sided 0.05. In addition, power calculation based on the log-rank test is provided as a comparison reference which will help inform the conclusions. Results are summarized in Fig. 1.

### Data Analysis

We take a subset of a completed and unblinded phase III CVOT dataset to demonstrate applications of these 3 approaches. The subset is defined by restricting to subjects who have been randomized no later than year 4 (i.e., 4 years from the first randomization) and by shortening the study duration to 4 years (i.e., by applying a common censoring data cut of 4 years from the first randomization). In addition to the 3 approaches, we further explore jump to reference (j2r) imputation, and a regular CPH model and use them as comparison basis.

Within this snapshot there are a total of 7740 subjects (5143 on the active treatment group and 1998 on placebo) in this dataset, among which 1362 are RDs, 776 are premature discontinuations and 466 are considered as missing data. These characteristics are summarized in Table 1. The primary endpoint is time to first occurrence of 3-point MACE defined as a composite endpoint of non-fatal stroke, non-fatal MI and CV death. There are 599 first MACE events (379 in active vs 220 in placebo) without imputation. We summarize the *n*(%) of events and censored prior to imputation by treatment group in Table 1. Missing data, RDs and completers are three mutually exclusive components of the dataset, adding up to 100%. For subjects in the category of “Have events prior to discontinuation”, if they are completely on-treatment leading to the first occurrence of MACE, they are considered completers; otherwise, they are considered RDs.

**Table 1:** Disposition of Subjects in the CVOT Snapshot

	Active	Placebo
Randomized	5143	2598
Discontinued the study	501 (9.7%)	275 (10.6%)
**Missing data**	**306 (5.9%)**	**160 (6.2%)**
Have events prior to discontinuation	195 (3.8%)	115 (4.4%)
**RDs**	**835 (16.2%)**	**527 (20.3%)**
Completers*	**4002 (77.8%)**	**1911 (73.6%)**

*Still in the study being followed by the snapshot date or have discontinued the study due to death

The 3 categories (Missing data, RDs and Completers) add up to the randomized population

*J2r approach* assumes the survival function of the active group will follow the distribution of the placebo group (coded as 0 without loss of generality) following study discontinuation. In other words, formula (1) will be adapted to reflect the distribution switch for missing data in active treatment group (coded as 1), written as (where $t>c$)

8 $$P\left((T\le t|T>\mathrm{c})|trt=1,\boldsymbol{ }{\varvec{X}}\right)=\frac{P\left(c<T\le t|trt=1,\boldsymbol{ }{\varvec{X}}\right)}{P\left(T>\mathrm{c}|trt=1,\boldsymbol{ }{\varvec{X}}\right)}=\frac{P\left(T>\mathrm{c}|trt=1,\boldsymbol{ }{\varvec{X}}\right)-P\left(T>t|trt=0,\boldsymbol{ }{\varvec{X}}\right)}{P\left(T>\mathrm{c}|trt=1,\boldsymbol{ }{\varvec{X}}\right)}=1-\frac{S\left(t|trt=0, X\right)}{S\left(c|trt=1, X\right)} $$

Then the estimate $\widehat{t}$ will be derived by solving the equation $\frac{S\left(t|trt=0, X\right)}{S\left(c|trt=1, X\right)}=u\sim U(\mathrm{0,1})$. In the multiple imputation setting ${{\varvec{\theta}}}^{(b)}$ in $\frac{S\left(t|trt=0, X; {{\varvec{\theta}}}^{(b)}\right)}{S\left(c|trt=1, X; {{\varvec{\theta}}}^{(b)}\right)}=u$ can be sampled using MCMC or MLE sampling using approximation or interpolation. In this case, we implement the J2R approach based on Weibull regression by means of MLE sampling because Weibull regression is considered as a substantive model [35, pp. 645–658]. The associated imputation formula is written as

9 $$\normalsize {{\widehat{t}}_{j}}^{(b)}=\mathrm{max}\left\{{\left(\mathrm{exp}\left(\frac{{\widehat{\eta }}_{j,0}^{\left(b\right)}-{\widehat{\eta }}_{j}^{\left(b\right)}}{{\widehat{\sigma }}^{\left(b\right)}}\right){{c}_{j}}^{\frac{1}{{\widehat{\sigma }}^{\left(b\right)}}}-\mathrm{exp}\left(\frac{{\widehat{\eta }}_{j,0}^{\left(b\right)}}{{\widehat{\sigma }}^{\left(b\right)}}\right)\mathrm{log}\left({{u}_{j}}^{\left(b\right)}\right)\right)}^{{\widehat{\sigma }}^{\left(b\right)}}, {c}_{j}\right\}$$

## Results

### Type-I Error

As shown in Table 2, all three approaches well control the type-I error rate and the bias relative to 0 is negligible (in log scale, 0 corresponding to HR = 1). It’s noteworthy in small sample size scenarios where the RDs and missing data are very imbalanced**,** the type-I error of piecewise exponential distribution seems a little deflated. For instance, when there are 5% RDs and 30% missing data in a study with a total of 1000 subjects, piecewise exponential distribution returns slightly deflated type-I error rate while the type-I error rate of the other two is close to 0.05 (Table 2).

**Table 2:** Type-I Error Results for Different Scenarios, Evaluated at a Two-Sided Alpha = 0.05

*N*	Different Proportion of RDs	Proportion of Discontinuation^#^	Piecewise Exponential		Bootstrap		Weibull	
			Type-I Error	Avg Bias*	Type-I Error	Avg Bias*	Type-I Error	Avg Bias*
1000	0.05	0.05	0.0494	0.005	0.0518	− 0.003	0.0352	− 0.002
	0.2	0.05	0.0532	− 0.001	0.0526	− 0.003	0.0518	− 0.003
	0.4	0.05	0.0528	− 0.002	0.0524	− 0.003	0.0524	0.002
	0.05	0.15	0.0386	0.018	0.0568	− 0.003	0.0128	− 0.005
	0.2	0.15	0.0532	0.003	0.0540	− 0.004	0.0506	− 0.004
	0.4	0.15	0.054	− 0.001	0.0550	− 0.004	0.0532	− 0.004
	0.05	0.3	0.0254	0.037	0.0526	− 0.005	0.0567	− 0.0002
	0.2	0.3	0.0529	0.010	0.0542	− 0.003	0.0464	− 0.003
	0.4	0.3	0.0551	0.003	0.0554	− 0.004	0.0534	− 0.004
5000	0.05	0.05	0.0538	0.002	0.0528	< 0.001	0.0526	< 0.001
	0.2	0.05	0.0514	0.001	0.0510	< 0.001	0.0506	< 0.001
	0.4	0.05	0.0526	< 0.001	0.0526	< 0.001	0.0528	< 0.001
	0.05	0.15	0.0488	0.005	0.0528	< 0.001	0.047	< 0.001
	0.2	0.15	0.0514	0.002	0.0514	< 0.001	0.0492	< 0.001
	0.4	0.15	0.0534	0.001	0.0520	< 0.001	0.0518	< 0.001
	0.05	0.3	0.0420	0.010	0.0492	− 0.0002	0.0356	< 0.001
	0.2	0.3	0.0516	0.002	0.0500	− 0.0003	0.0500	− 0.0002
	0.4	0.3	0.0514	0.001	0.0523	− 0.0004	0.0516	< 0.001
10,000	0.05	0.05	0.0536	0.002	0.0524	0.0015	0.0526	0.0015
	0.2	0.05	0.0516	0.002	0.0514	0.0015	0.0508	0.0015
	0.4	0.05	0.0510	0.002	0.0525	0.001	0.0514	0.0015
	0.05	0.15	0.0484	0.005	0.0482	0.002	0.0468	0.0020
	0.2	0.15	0.0506	0.002	0.0485	0.001	0.0490	0.0019
	0.4	0.15	0.0510	0.002	0.0510	0.0026	0.0504	0.0019
	0.05	0.3	0.0456	0.007	0.0470	0.0019	0.0424	0.0025
	0.2	0.3	0.0488	0.004	0.0478	0.0029	0.0496	0.0025
	0.4	0.3	0.0488	0.003	0.0470	0.0019	0.0492	0.0025

^#^Missing rate is lower than proportion of study discontinuation because subjects with events prior to study discontinuation are not counted as missing data

*On log(HR) scale

### Power Rate

All three approaches have very similar power rate, with the bootstrap approach slightly more powerful than the other two (Fig. 1). Furthermore, all three approaches follow similar patterns: (1) given a sample size and an RD rate, more missing data will lead to some power loss; (2) When sample size is large and given a study discontinuation rate, higher proportion of RDs results in a slightly higher power compared to studies with a lower proportion. (3) When sample size is small to medium, given a study discontinuation rate, low RD rates lead to higher power rates. It’s very noteworthy in some scenarios the proposed approaches are more powerful than log-rank test assuming no RDs in the study. Different competing forces that contribute to these phenomena will be discussed in the “Discussion” section.

**Figure 2:**
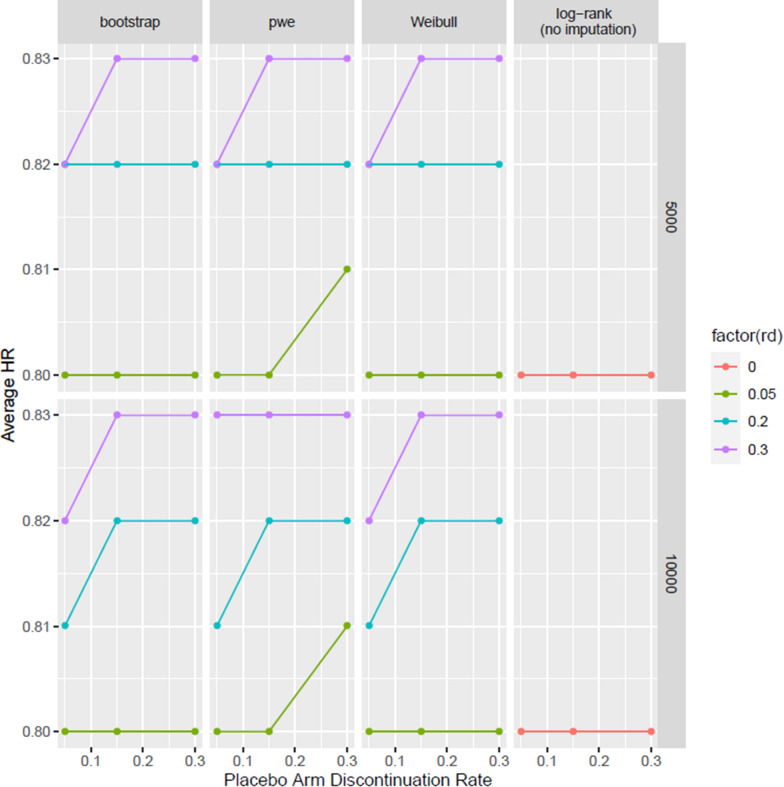
Compare the HR of Three MIRD Approaches Under Different Clinical Scenarios

From the perspective of hazard ratio, all three approaches lead to slightly reduced risk reduction (i.e. larger HR) after adjusting for diluted treatment effect contributed by RDs (Fig. 2). Within the same sub plot, more RDs lead to a more reduced risk reduction (i.e., a larger HR); for instance, the HR corresponding to 20% RDs is larger than the one with 5% RDs.

### Data Analysis

All imputation approaches lead to an increase in number of events, compared to the regular CPH. Among all approaches, J2R leads to the highest increase in number of events, especially in the active group which explains why its estimated risk reduction (= 1 − HR) is the smallest of all. All three approaches yield very similar results consistently, with risk reduction all larger than J2R but slightly smaller than CPH. For the bootstrap version, using more refined grids will lead to an estimated risk reduction very similar to the CPH, according to Table 3.

**Table 3:** Results of Using Different Statistical Methods to Analyze Time to First MACE

Method	Events in placebo	Events in Active	HR (95% CI) vs Placebo	*p*-Value
cph	220	379	0.86 (0.73, 1.02)	0.08
j2r	224.2	392.8	0.87 (0.74, 1.03)	0.11
MIRD_weibull	222.5	385.8	0.86 (0.73, 1.02)	0.09
MIRD_pwe*	222.4	386.9	0.87 (0.73, 1.02)	0.09
MIRD_bootstrap (grid width = 60 days)	222.5	385.4	0.86 (0.73, 1.02)	0.08
MIRD_bootstrap (grid width = 90 days)	222.4	386.9	0.87 (0.73, 1.02)	0.09

*4 piecewise exponential hazards are used with cut points generated using cuts < -c(0, quantile(A$t2e)[2:4], max(A$t2e) + 1) before passed to the piecewise function

## Software Implementation

SAS is recommended for implementation of MCMC sampling because Bayesian sampling is readily embedded in several relevant SAS procedures such as Proc Lifereg, Proc MCMC. In combination with these SAS procedures, it’s adequate to use SAS for imputation and data analyses. For example, users can firstly use Proc lifereg to generate the Bayesian sampling of the model parameters, and then perform matrix manipulation through Proc IML for time to events imputation, followed by Proc Phreg for the final analysis by imputation. Nevertheless, overall, it still poses some computing challenges for simulations, especially for approaches involving intensive interpolations and sampling. R is used in all simulations of this manuscript with the use of MLE sampling, due to the asymptotic equivalence [36, pp. 473–483]. The only limitation exists in piecewise exponential imputation because the current R package “eha” only provides a partial covariance matrix estimate, restricting to regression coefficients only (i.e. without piecewise baseline hazards). All R functions and SAS macros are provided in the supplementary files for reference.

## Discussion

Cox proportional hazards model has long been regarded as the gold standard to analyze time to first event endpoints in cardiovascular and renal outcome trials [37, pp. 1425–1435, 38, pp. 896–907, 39, pp. 1436–1446, 40, pp. 841–851, 41, pp. 2295–2306, 5, pp. 1527–1539, 42, pp. 347–357, 43, pp. 2117–2128]. On one hand, it’s still frequently used as primary analysis as a well-established model. On the other hand, it has been noted that censoring for premature study discontinuation, known as presumed “non-informative censoring” can result in potential bias in the treatment effect estimate across different therapeutic areas [44, pp. 2001–2009, 10, p. 40, 45, p. 101865, 9, p. 4, 46, pp. 327–328, 47, pp. 1433–1440]. In our proposed analyses, subjects censored due to non-administrative reasons are treated as missing data if they don’t have relevant events prior to the discontinuation, and the potential for informative censoring is allowed by imputing their outcome assuming they follow the distribution of RDs in the same randomized group. Generally, they are not considered as missing due to random reasons, and we aim to impute their outcome after their study discontinuation, based on the assumption that their time to event distribution will approximately follow the distribution of RDs in the same randomized group. As a MNAR assumption, this serves as the foundation for implementation of the imputation. The subsequent analysis and combining steps are common to almost all MI approaches.

Multiple imputation is favored over single imputation because it allows uncertainty by means of multiple sampling, regardless of whether the sampling is parametric or not. Furthermore, it leads to non-biased estimates by using Rubin’s rule which combines within and between imputation variability [29, pp. 202–243]. Unlike the continuous MI-RD approach, implementation of time to event MI-RD approach doesn’t have small sample size issues in CVOT. Because size of outcome trials is normally large and usually it has more RDs than missing data, there is a high degree of plausibility that the model-based standard error is sufficiently small to get imputation values that closely approximate the results that would have been obtained in the real world. Generally speaking, 5–20% RDs in a CVOT will enable the implementation of the MI-RD approach.

All three variations have well-controlled type-I error rate. In terms of power rate, there are scenarios under which these approaches lead to improved power rate, as a result of interactions of two “competing” forces. The first one refers to increase in number of events from imputation, while the other one refers to attenuated risk reduction (i.e., larger HR) after imputation. More specifically, these MI approaches based on RDs tend to increase the overall hazard rate of the active group post-imputation due to a slightly increased hazard rate in the active group during the off-treatment period. Time to event endpoints are mainly powered by total number of events and the alternative HR assumption. The first one has the potential to increase power via the additional events added by imputation while the latter has the tendency to reduce power via attenuation of treatment effect toward an HR that is closer to 1.0. The scenarios in which the first force outweigh the 2nd one are those with power gain over traditional log-rank tests assuming no RDs (Fig. 1). In the setting of a typical superiority CVOT with 4000 to 10,000 randomized subjects, 15–25% RD rate and 1% annualized study discontinuation rate, our proposed approaches will lead to increased power rates. For smaller CVOTs (first two rows in Fig. 1), more RDs lead to reduced power rates in every sub plot because the impact of treatment effect attenuation outweighs increase in events post-imputation.

The proposed three approaches can be implemented and applied to real clinical datasets in either R or SAS on PC platform. They have very similar performance but slightly different computational complexity. For each approach, users can either choose MCMC sampling or MLE sampling because they are asymptotically equivalent. Although the current R implementation for piecewise exponential distribution using R package “eha” only allows partial sampling of parameters, the type-I error and power rate assessments do align with the results of other approaches. Users can find all R functions and SAS examples in the supplementary files. No matter which approach the sponsor pre-specifies for the clinical study, it’s essential to gain consensus with the health authorities (HA) before unblinding a study.

In this manuscript, we propose an example of using a MNAR assumption to impute missing data of time to event endpoints. There are various ways to pre-specify and to perform sensitivity analyses. The fundamentals are to decide which assumption best fits the purpose. A conservative assumption has the potential to solidify the primary conclusion if the results don’t alter the primary conclusion, but it also has the risk of deviating from primary results. Because our assumption is neither conservative nor aggressive, it should be relatively acceptable to both HA and sponsors.

## Conclusion

We propose three MI approaches based on the same MNAR assumption for time to event endpoints in CVOT. They truly are estimation approaches that can be implemented using non-parametric bootstrap or parametric methods via either MCMC or MLE multivariate sampling (“proper imputations”) due to asymptotic equivalence. These approaches can be readily extended to studies in other therapeutic areas if the trials continue to follow patients regardless of treatment discontinuation or not. The three proposed approaches have very similar type-I error and power rates given a clinical scenario, with bootstrap being the most optimal solution due to the nature of the approach that it takes into account uncertainty by not imposing parametric restriction. It’s noteworthy that bootstrap approach is more computationally intensive, while Weibull regression is the least. When bootstrap computation is a burden, Weibull or piecewise regression will suffice according to the supplementary analysis conducted: These two approaches are also robust when the underlying data deviates from the parametric distribution. Because the underlying assumption is not as conservative as jump to reference multiple imputation, the estimated HR usually is smaller than jump to reference analyses but larger than Cox model. We believe the proposed MI approaches meet the expectation of health authorities in terms of their capabilities to justify robustness of primary conclusions. Furthermore, they can serve as primary analysis and driver of sample size estimation.

### Supplementary Information

Below is the link to the electronic supplementary material.

Supplementary file1 (DOCX 44 KB)

## Data Availability

To protect the patients' privacy, the real CVOT dataset will not be accessible to the public.
