# Supplementary material for: Imputation of Missing Data for Time-to-Event Endpoints Using Retrieved Dropouts
Source: Ther Innov Regul Sci. 2023 Oct 7;58(1):114–26. doi: 10.1007/s43441-023-00575-5 (PMC10764582; doi:10.1007/s43441-023-00575-5)
Supplement: Supplementary file 1 — Supplementary file1 (DOCX 44 KB) [file 43441_2023_575_MOESM1_ESM.docx]

# Supplementary tables

**Supplementary Table 1. Type-I error results for times to event simulated using uniform distribution.**

| N | Different proportion of RDs | Proportion of discontinuation^#^ | Piecewise exponential | | Bootstrap | | | Weibull | |
| --- | --- | --- | --- | --- | --- | --- | --- | --- | --- |
|  |  |  | Type-I error | Avg bias* | Type-I error | | Avg bias* | Type-I error | Avg bias* |
| 1000 | 0.05 | 0.05 | 0.048 | -0.003 | 0.050 | -0.003 | | 0.033 | <-0.001 |
|  | 0.2 | 0.05 | 0.050 | -0.002 | 0.051 | -0.003 | | 0.0516 | -0.003 |
|  | 0.4 | 0.05 | 0.051 | -0.002 | 0.054 | -0.003 | | 0.0528 | -0.003 |
|  | 0.05 | 0.15 | 0.053 | <0.001 | 0.053 | -0.004 | | 0.013 | -0.003 |
|  | 0.2 | 0.15 | 0.045 | -0.003 | 0.053 | -0.004 | | 0.0496 | -0.004 |
|  | 0.4 | 0.15 | 0.050 | -0.003 | 0.053 | -0.004 | | 0.0512 | -0.004 |
|  | 0.05 | 0.3 | 0.071 | 0.002 | 0.058 | -0.003 | | 0.0043 | 0.001 |
|  | 0.2 | 0.3 | 0.035 | -0.004 | 0.053 | -0.005 | | 0.0448 | -0.005 |
|  | 0.4 | 0.3 | 0.045 | -0.004 | 0.055 | -0.005 | | 0.0524 | -0.005 |
| 5000 | 0.05 | 0.05 | 0.051 | <0.001 | 0.053 | <0.001 | | 0.0506 | <0.001 |
|  | 0.2 | 0.05 | 0.054 | <0.001 | 0.054 | <0.001 | | 0.0542 | <0.001 |
|  | 0.4 | 0.05 | 0.053 | <0.001 | 0.052 | <0.001 | | 0.0522 | <-0.001 |
|  | 0.05 | 0.15 | 0.034 | <0.001 | 0.048 | <0.001 | | 0.043 | <0.001 |
|  | 0.2 | 0.15 | 0.049 | <0.001 | 0.051 | <0.001 | | 0.0508 | <0.001 |
|  | 0.4 | 0.15 | 0.049 | <0.001 | 0.051 | <0.001 | | 0.0498 | <0.001 |
|  | 0.05 | 0.3 | 0.025 | <-0.001 | 0.052 | -<0.001 | | 0.0378 | <-0.001 |
|  | 0.2 | 0.3 | 0.043 | <-0.001 | 0.051 | -<0.001 | | 0.0512 | <-0.001 |
|  | 0.4 | 0.3 | 0.049 | <-0.001 | 0.050 | -<0.001 | | 0.0504 | <-0.001 |
| 10000 | 0.05 | 0.05 | 0.048 | 0.0016 | 0.052 | 0.002 | | 0.0502 | 0.0016 |
|  | 0.2 | 0.05 | 0.052 | 0.0016 | 0.052 | 0.0015 | | 0.0508 | 0.0015 |
|  | 0.4 | 0.05 | 0.051 | 0.0015 | 0.052 | 0.0015 | | 0.0516 | 0.0015 |
|  | 0.05 | 0.15 | 0.044 | 0.0014 | 0.052 | 0.0017 | | 0.0484 | 0.0017 |
|  | 0.2 | 0.15 | 0.049 | 0.0018 | 0.051 | 0.0019 | | 0.0498 | 0.0019 |
|  | 0.4 | 0.15 | 0.048 | 0.002 | 0.049 | 0.0020 | | 0.0502 | 0.0020 |
|  | 0.05 | 0.3 | 0.036 | <0.001 | 0.046 | 0.0021 | | 0.0426 | 0.0022 |
|  | 0.2 | 0.3 | 0.044 | 0.004 | 0.049 | 0.0025 | | 0.0484 | 0.0027 |
|  | 0.4 | 0.3 | 0.049 | 0.004 | 0.045 | 0.0023 | | 0.0496 | 0.0025 |

^#^ : missing rate is lower than proportion of study discontinuation because subjects with events prior to study discontinuation are not counted as missing data.

*: on log(HR) scale.

# SAS and R code:

## MIRD using Weibull regression

### SAS Implementation

*SAS implementation*:

data demo;
do i=1 to 4000;/* simulate 4000 subjects */
if i>500 then trt=1;
else trt=0;
randdt=rand('UNIFORM',0,90);
datacut=2*365;
t2e=rand('UNIFORM', randdt, datacut)-randdt;
event=rand('BERNOULLI', 0.05);
if event=0 then t2e=datacut-randdt;
dis=rand('BERNOULLI', 0.1);
if dis=0 then do;
rd=rand('BERNOULLI', 0.15);
imp=0;
end;
else do;
t2d=rand('UNIFORM', randdt, datacut)-randdt;
if (t2e>t2d) and (event=1) then do;
    event=0;
    t2e=t2d;
end;
rd=0;
if (dis=1) and (event=0) then imp=1;
end;
output;
end;
run;

Options symbolgen mlogic mprint;

%include "/home/wangs159/MIRD_Surv_DA/MIRD_weibull.sas";

%weibull_mird(data=demo, nimpute=100,

rd_flag=(rd=1), trt=trt, impute_flag=(imp=1), nonimpute_flag=(imp=0), t2e=t2e, time_start=randdt, cutoff=730, status_mace=event, cnsrval=0, eventval=1, miseed=123, normalseed=999, nthin=50);

;

*below is the MIRD_weibull SAS macro;

/*

**** Author: Shuai Wang (Shuai.Wang6@pfizer.com or shuai1107@hotmail.com)

**** Date: Apr 16, 2023

************************************************************************************************************

************************************************************************************************************

************************************************************************************************************

**** Input Arguments **************************************************************************************

**** data: data set name with two prenamed columns (trt: 1=active,0=placebo; vital_status: alive_value denotes alive, otherwise dead)

**** nimpute: number of imputations

**** rd_flag: flag used to select the restrieved dropouts (1=retrieved, 0=not retrieved)

**** impute_flag: flag used to select subjects that discontinue the trial and the time to event is unknown even with the information of vital status

* nonimpute_flag: as opposed to impute_flag: subjects whose time to event is known and doesn't need imputation

**** t2e: time to first event variable

**** cnsrval: numeric censor value

**** eventval: numeric event value

**** time_start: the variable that shows when each subject enrolled in the study (relative to the first randomization for instance)

**** cutoff: the study common cutoff date (relative to the first day)

**** status_mace: event/censor indicator

**** miseed: used to generate the parameter estimate, weibull parameter from the posterior distribution

**** normalseed: used to generate the radom number from uniform(0,1)

**** nthin: controls the thinning of the Markov chain. Only one in every k samples is used when nthin=k

*/

%macro weibull_mird(data=, nimpute=,

rd_flag=, trt=, impute_flag=, nonimpute_flag=, t2e=, cnsrval=, eventval=, time_start=, cutoff=, status_mace=, miseed=, normalseed=, nthin=);

%let total_nmc=%sysevalf(&nimpute*&nthin);

%put &total_nmc;

proc lifereg data=&data (where=(&rd_flag)) covout outest=covest ;

class &trt;

model &t2e*&status_mace(&cnsrval)=&trt /dist=weibull;

bayes seed=&miseed coeffprior=normal nmc=&total_nmc thin=&nthin;

ods output posteriorsample=ps;

run;

data ps;

set ps;

_imputation_=iteration-2000;/*use the default # of burn-in iteration*/

run;

/*proc lifereg data=&data (where=(&rd_flag)) covout outest=covest ;

model &time2nfm*&status_nfm(0)=trt /dist=weibull;

bayes seed=&miseed coeffprior=normal nmc=&total_nmc thin=&nthin;

ods output posteriorsample=ps_nfm;

run;

data ps_nfm;

set ps_nfm;

_imputation_=iteration-2000;

run;*/

proc iml;

Nimpute=&nimpute;

use &data;

read all var{&trt &t2e &time_start &status_mace } where (&impute_flag) into ximp;

read all var{&trt &t2e &time_start &status_mace } where (&nonimpute_flag) into xnonimp;

close &data;

N_imp=nrow(ximp);

N=N_imp+nrow(xnonimp);

*print mean covariance;

*print ximp;

create data_imp var{&trt &t2e &time_start &status_mace _imputation_} ;

do i = 1 to Nimpute;

xmatrix=ximp;

mattrib xmatrix colname={&trt &t2e &time_start &status_mace };

use ps;

read all where (_imputation_=i) into coef;

close ps;

print coef;

beta0=coef[2];

beta_1=coef[3];

sigma=coef[5];

gamma=1/sigma;

mu=beta0*repeat({1}, N_imp)+beta_1*xmatrix[,1];

sel_act=loc(xmatrix[,1]=1);

mu[sel_act]=beta0*repeat({1}, ncol(sel_act));

*generate the random numbers from uniform distribution U(0,1);

u_seed=j(N_imp, 1, &normalseed);

u=uniform(u_seed);

t_imputed=xmatrix[,2]+(-log(u)#exp(mu#gamma))##sigma;

print t_imputed;

xmatrix[,4]=&eventval;/*&eventval is the event value*/

censor=loc(xmatrix[,3]+t_imputed>&cutoff);

if ncol(censor)>0 then xmatrix[censor,4]=&cnsrval;

xmatrix[,2]=t_imputed><(&cutoff-xmatrix[,3]);

xdata=(xnonimp//xmatrix)||repeat(i,N,1);

mattrib xdata colname={&trt &t2e &time_start &status_mace _imputation_};

append from xdata;

end;

close data_imp;

quit;

/*data data_imp;

set data_imp;

if &trt=3 then &trt=2;

run;*/

proc phreg data=data_imp;

by _imputation_;

class &trt(ref="0") ;

model &t2e*&status_mace(&cnsrval)=&trt;

contrast "active vs placebo" &trt 1 /estimate=parm;

ods output ContrastEstimate=est;

run;

proc sort data=est;

by contrast;

run;

proc mianalyze data=est;

by contrast;

modeleffects estimate;

stderr stderr;

ods output parameterestimates=est_mi;

run;

data est_mi2;

set est_mi;

sup_stats=-abs(estimate)/stderr;

p_sup=cdf("t",sup_stats , df)*2;

HR=exp(estimate);

lowCI=exp(lclmean);

upperCI=exp(uclmean);

run;

proc print data=est_mi2;

run;

%mend;

### R implementation

set.seed(123)

N<-4000

subj<-1:N

trt<-rep(c(0,1), each=N/2)

randdt<-runif(N, 0, 90)

datacut<-2*365

t2e<-sapply(randdt, function(x){runif(1, x, datacut)-x})

event<-rbinom(N, 1, 0.05);

#for censoring, set to datacut-randomization date;

t2e[event==0]<-datacut-randdt[event==0]

dis<-rbinom(N, 1, 0.1);

rd<-rep(0, N)

imp<-rep(0, N)

#randomly select rds among subjects that stay in the trial;

rd[dis==0]<-rbinom(sum(dis==0), 1, 0.15)

#for subjects who stay in the trial they are not regarded as missing data;

imp[dis==0]<-0

t2d<-datacut-randdt

#simulate time to discontinuation for dis=1;

t2d[dis==1]<-sapply(randdt[dis==1], function(x){runif(1, x, datacut)-x})

#for subjects whose time to event occurs later than time to discontinuation, they will be censored;

cnsr.cond<-which((t2e>t2d)& (event==1)& (dis==1) )

event[cnsr.cond]<-0

t2e[cnsr.cond]<-t2d[cnsr.cond]

rd[dis==1]<-0;

#subjects who discontinue without events are missing data;

imp.cond<- which((dis==1) & (event==0))

imp[imp.cond]<-1

A<-data.frame(subj, trt, randdt, datacut=rep(2*365, 1000), t2e, event, dis, rd, imp)

#The function below requires installation of package "SurvRegCensCov"

res<-weibull.cph(data=A , B=100, Time="t2e" , Rdfl="rd==1", Impfl="imp==1", datacutvar="datacut", startvar="randdt", status="event", statusvalue=1, trtvar="trt", formula=~trt, formula_var="trt", seed=123)

#Below code shows how to combine all B results into a single estimate (It also applies to 2.2 and 2.3);

beta=mean(res[,1])

var_w=mean(res[,2])

var_b=var(res[,1])

var_oa=var_w+(1+1/100)*var_b;

test_stats=beta/sqrt(var_oa);

df=(100-1)*(1+var_w/(1+1/100)/var_b)^2;

p.wbmird=pt(abs(test_stats), df=df, lower.tail=F)*2;

##Below function uses Weibull distribution to multiply impute time to event missing data. Assume there are two treatment groups, a B by 4 matrix will be generated in the output, with the columns in the order of 1) HR in log scale, 2) Variance, 3) and 4) average number of events in the two groups respectively;

weibull.cph<-function(data , B=100, Time , Rdfl, Impfl, datacutvar, startvar, status,statusvalue, trtvar, formula, formula_var, seed){

#library(gtools);

library(survival);

library("SurvRegCensCov")

library(MASS);

RDdata=subset(data, eval(parse(text=Rdfl)))

IMPdata=subset(data, eval(parse(text=Impfl)))

#Nboot<-t(sapply(1:B, function(x){sample(1:N, N, replace=T)}))

formul<-paste("Surv(", Time, "," , status, ")", paste(as.character(formula), collapse = ""), sep="");

formul<-as.formula(formul);

coxreg<-WeibullReg(formula=formul, data=RDdata)

coefs=coxreg$summary$table[,1]

covarmat=coxreg$summary$var

set.seed(seed)

coefs.draw=mvrnorm(n = B, mu=coefs, Sigma=covarmat, tol = 1e-6, empirical = FALSE, EISPACK = FALSE)

res.draw=apply(coefs.draw, 1, function(x){

p.rand=runif(nrow(IMPdata))

coef_draw=x[-length(x)]

scale=exp(tail(x,1))

gamma=1/scale

#SurvProb.rand=SurvProb.Imp*p.rand #use them to estimate time to event after study discontinuation;

eta=as.matrix(cbind(rep(1, nrow(IMPdata)), IMPdata[, formula_var]))%*%matrix(coef_draw, ncol=1)

imp.t=(IMPdata[, Time]^gamma-exp(eta/scale)*log(p.rand))^scale

imp.t=unlist(imp.t)

imp.t[is.infinite(imp.t)]=999999

IMPdata.draw=IMPdata

IMPdata.draw[, status]=ifelse(imp.t+IMPdata.draw[, startvar]>IMPdata.draw[, datacutvar], 0, 1)

IMPdata.draw[, Time]=apply(cbind(IMPdata.draw[, c(status, datacutvar, startvar)], imp.t), 1, function(x){

if (x[1]==1){

return(x[4])

} else{

return(x[2]-x[3])

}

})

#analyze the complete dataset;

data.draw=rbind(subset(data, eval(parse(text=paste("!", Impfl, sep="")))), IMPdata.draw)

#data.draw[,Time]=as.numeric(data.draw[,Time])

#data.draw[, status]=as.numeric(data.draw[, status])

coxreg.draw=coxph(formul, data=data.draw)

coefs_all=coef(coxreg.draw)

variance.all=coxreg.draw$var

#eval(parse(text=paste(status, "==", statusvalue, sep="")))

EventTable<-table(subset(data.draw, eval(parse(text=paste(status, "==", statusvalue, sep=""))), select=trtvar))

return(c(coefs_all, variance.all, EventTable))

})

res.draw=t(res.draw)

#EventTable<-table(subset(data, status==statusvalue, select="trt"))

colnames(res.draw)[1:2]=c("beta", "variance")

colnames(res.draw)[-c(1:2)]=paste(trtvar, "_", colnames(res.draw)[-c(1:2)], sep="")

return(res.draw)

}

## MIRD using piecewise exponential

cuts<-c(0, quantile(A$t2e)[2:4], max(A$t2e)+1)

#The function below requires installation of package "eha"

Res<-pwe.cph(data=A, B=100, Time="t2e", Rdfl="rd==1", Impfl="imp==1", datacutvar="datacut", startvar="randdt", status="event", statusvalue=1, trtvar="trt", formula=~trt, seed=456, cuts=cuts)

##Below function uses Piecewise exponential to multiply impute time to event missing data. Assume there are two treatment groups, a B by 4 matrix will be generated in the output, with the columns in the order of 1) HR in log scale, 2) Variance, 3) and 4) average number of events in the two groups respectively;

pwe.cph<-function(data , B=100, Time , Rdfl, Impfl, datacutvar, startvar, status, statusvalue, trtvar, formula, seed, cuts){

#library(gtools);

library(survival);

library(eha);

library(MASS);

#library("dplyr")

RDdata=subset(data, eval(parse(text=Rdfl)))

IMPdata=subset(data, eval(parse(text=Impfl)))

N<-nrow(RDdata);

#Nboot<-t(sapply(1:B, function(x){sample(1:N, N, replace=T)}))

formul<-paste("Surv(", Time, "," , status, ")", paste(as.character(formula), collapse = ""), sep="");

formul<-as.formula(formul);

coxreg<-pchreg(formula=formul, data=RDdata, cuts=cuts)

coef.mle=coxreg$coefficients

var.mle=coxreg$var

set.seed(seed)

N_hint=length(coxreg$hazards)

coefs.draw=mvrnorm(n = B, mu=as.numeric(coef.mle), Sigma=var.mle, tol = 1e-6, empirical = FALSE, EISPACK = FALSE)

res.draw=apply(coefs.draw, 1, function(x){

beta=matrix(x, ncol=1)

lambda=coxreg$hazards

IMPdata2=data.frame(IMPdata, subj=1:nrow(IMPdata))

#fit the survival on censored timepoint for imputed population;

hazards_m_interval=diff(coxreg$cuts)*coxreg$hazards

SurvProb.Imp=by(IMPdata2, IMPdata2$subj, function(z){

N_int=findInterval(z[Time], coxreg$cuts)

x_covar=z[coxreg$covars]

eta=as.matrix(x_covar)%*%beta;

if (N_int<1 | N_int>=length(coxreg$cuts)){

stop ("some observation doesn't fall in the cut point intervals!")

} else if (N_int==1){

S=exp(-lambda[1]*(z[Time]-coxreg$cuts[1])*exp(eta))

} else{

cum_lambda_int=lambda[N_int]*(z[Time]-coxreg$cuts[N_int])+sum(hazards_m_interval[1:(N_int-1)])

S=exp(-cum_lambda_int*exp(eta))

}

return(S)

})

SurvProb.Imp=do.call(c, SurvProb.Imp)

#SurvProb.Imp=predict(coxreg.draw, IMPdata, type="distr")$Surv

p.rand=runif(nrow(IMPdata))

SurvProb.rand=SurvProb.Imp*p.rand #use them to estimate time to event after study discontinuation;

IMPdata2=data.frame(IMPdata, p=SurvProb.rand, subj=1:nrow(IMPdata))

imp.t=by(IMPdata2, IMPdata2$subj, function(z){

#generate survival probability for cutpoints for each imputation subj;

x_covar=z[coxreg$covars]

eta=as.matrix(x_covar)%*%beta;

surv_cut=sapply(1:(N_hint-1), function(m){

return(exp(-sum(hazards_m_interval[1:m])*exp(eta)))

})

surv_cut=c(0, sort(surv_cut), 1)

N_int=findInterval(z$p, surv_cut)

if (N_int==1) {

t_imp=coxreg$cuts[N_hint]-(log(z$p)*exp(-eta)+sum(hazards_m_interval[1:(N_hint-1)]))/lambda[N_hint]

} else if (N_int==N_hint) {

t_imp=-log(z$p)*exp(-eta)/lambda[1]

} else {

t_imp=coxreg$cuts[N_hint-(N_int-1)]-(log(z$p)*exp(-eta)+sum(hazards_m_interval[1:(N_hint-N_int)]))/lambda[N_hint-N_int+1]

}

return(t_imp)

})

imp.t[is.infinite(imp.t)]=999999

imp.t=do.call(c, list(imp.t))

IMPdata.draw=IMPdata

IMPdata.draw[, status]=ifelse(imp.t+IMPdata.draw[, startvar]>IMPdata.draw[, datacutvar], 0, 1)

#IMPdata.draw[, Time]=ifelse(IMPdata.draw[, status]==1, imp.t, IMPdata.draw[, datacutvar]-IMPdata.draw[, startvar])

IMPdata.draw[, Time]=apply(cbind(IMPdata.draw[, c(status, datacutvar, startvar)], imp.t), 1, function(x){

return(ifelse(x[1]==1, x[4], x[2]-x[3]))})

#analyze the complete dataset;

data.draw=rbind(subset(data, eval(parse(text=paste("!", Impfl, sep="")))), IMPdata.draw)

data.draw[,Time]=as.numeric(data.draw[,Time])

data.draw[, status]=as.numeric(data.draw[, status])

coxreg.draw=coxph(formul, data=data.draw)

coefs.draw=coef(coxreg.draw)

variance.draw=coxreg.draw$var

EventTable<-table(subset(data.draw, eval(parse(text=paste(status, "==", statusvalue, sep=""))), select=trtvar))

return(c(coefs.draw, variance.draw, EventTable))

})

res.draw=t(res.draw)

#EventTable<-table(subset(data, status==statusvalue, select="trt"))

colnames(res.draw)[1:2]=c("beta", "variance")

colnames(res.draw)[-c(1:2)]=paste(trtvar, "_", colnames(res.draw)[-c(1:2)], sep="")

return(res.draw)

}

## MIRD using bootstrap

#In below example because time to event is in days, grid_w=30 means the distance between two adjacent grids is 30 days;

#Calling below function requires installation of “gtools” and “rapportools”;

Res<- boot.cph(data=A, B=100, subjid="subj", Time="t2e" , Rdfl="rd==1", Impfl="imp==1", datacutvar="datacut", startvar="randdt", status="event", statusvalue=1, trtvar="trt", formula=~trt, seed=123, grid_w=30)

## Below function uses boostrap sampling to multiply impute time to event missing data. Assume there are two treatment groups, a B by 4 matrix will be generated in the output, with the columns in the order of 1) HR in log scale, 2) Variance, 3) and 4) average number of events in the two groups respectively;

boot.cph<-function(data , subjid, B=100, Time , Rdfl, Impfl, datacutvar, startvar, status, statusvalue, trtvar, formula, seed, grid_w){

library(gtools);

library(survival);

library("dplyr")

library(rapportools)

RDdata=subset(data, eval(parse(text=Rdfl)))

IMPdata=subset(data, eval(parse(text=Impfl)))

N<-nrow(RDdata);

Nboot<-t(sapply(1:B, function(x){sample(1:N, N, replace=T)}))

set.seed(seed)

res.boot=apply(Nboot, 1, function(x){

data.tmp<-RDdata[x,];

formul<-paste("Surv(", Time, "," , status, ")", paste(as.character(formula), collapse = ""), sep="");

formul<-as.formula(formul);

coxreg<-coxph(formul, data=data.tmp)

SurvProb.Imp=predict(coxreg, IMPdata, type="survival")

p.rand=runif(nrow(IMPdata))

SurvProb.rand=SurvProb.Imp*p.rand #use them to estimate time to event after study discontinuation;

IMPdata_aug=data.frame(IMPdata, SurvProb.rand)

IMPdata_post=by(IMPdata_aug, IMPdata_aug[, subjid], function(z){

#determine how many interpolation points;

start=as.numeric(z[Time])

end=as.numeric(z[datacutvar])-as.numeric(z[startvar])

n_interp=round((end-start)/grid_w)+1

z.extrop=do.call(rbind, replicate(n_interp, z, simplify = FALSE))

z.extrop[, Time]=c(seq(start, by=grid_w, length.out=(n_interp-1)), end)

#z.extrop=data.frame(z.extrop)

# z.extrop=lapply(z.extrop, unfactor)

# z.extrop=do.call(data.frame, z.extrop)

# z.extrop[,Time]=as.numeric(z.extrop[,Time]);

# z.extrop[,status]=as.numeric(z.extrop[,status]);

survprob.extrop=predict(coxreg, z.extrop, type="survival")

z.survprob=as.numeric(z[ "SurvProb.rand"])

start.ind=tail(which(survprob.extrop>z.survprob),1)

end.ind=which(survprob.extrop<z.survprob)[1]

if (is.empty(start.ind)|is.empty(end.ind)){

z[ status]=0

z[ Time]=end

} else{

#interpolation;

start.p=survprob.extrop[start.ind]

start.time=z.extrop[start.ind, Time]

end.p=survprob.extrop[end.ind]

end.time=z.extrop[end.ind, Time]

slope=(end.time-start.time)/(end.p-start.p)

imp.time=start.time+(z.survprob-start.p)*slope

z[ status]=ifelse(imp.time>end, 0, 1)

z[ Time]=ifelse(z[ status]==1, imp.time, end)

}

return(z)

})

IMPdata_post=do.call(rbind, IMPdata_post);

#analyze the complete dataset;

data_boot=rbind(subset(data, eval(parse(text=paste("!", Impfl, sep="")))), IMPdata_post[, -ncol(IMPdata_post)])

data_boot[,Time]=as.numeric(data_boot[,Time])

data_boot[, status]=as.numeric(data_boot[, status])

reg_boot=coxph(formul, data=data_boot)

coefs.boot=coef(reg_boot)

variance.boot=reg_boot$var

EventTable<-table(subset(data_boot, eval(parse(text=paste(status, "==", statusvalue, sep=""))), select=trtvar))

return(c(coefs.boot, variance.boot, EventTable))

})

res.boot=t(res.boot)

colnames(res.boot)[1:2]=c("beta", "variance")

colnames(res.boot)[-c(1:2)]=paste(trtvar, "_", colnames(res.boot)[-c(1:2)], sep="")

return(res.boot)

}
